# Designing an integrated blockchain-enabled supply chain network under uncertainty

**DOI:** 10.1038/s41598-023-30439-9

**Published:** 2023-03-09

**Authors:** Ardavan Babaei, Majid Khedmati, Mohammad Reza Akbari Jokar, Erfan Babaee Tirkolaee

**Affiliations:** 1 Department of Industrial Engineering, Sharif University of Technology, Tehran, Iran; 2 Department of Industrial Engineering, Istinye University, Istanbul, Turkey

**Keywords:** Computational science, Information technology

## Abstract

With the development of communication infrastructure, the design of supply chains has changed significantly. Blockchain technology, as one of the most cutting-edge technologies, can promote transparency among members of the supply chain network. To the best of our knowledge, this is the first study that tries to develop a novel bi-objective optimization model to integrate the transparency resulting from the use of blockchain for designing a three-level supply chain network. The first objective function is to minimize total cost while the second objective function seeks to maximize transparency based on the application of blockchain technology. Moreover, it is worth noting that it is the first attempt to investigate the role of a blockchain model under stochastic conditions. The bi-objectiveness and stochastic nature of the proposed model are then treated using Fuzzy Goal Programming (FGP) and Chance-Constrained programming (CCP) approaches, respectively. To tackle the problem, an improved Branch and Efficiency (B&E) algorithm is developed by incorporating transparency along with cost and service. The impacts of blockchain exclusively through transparency (Case 1) or through transparency, cost, and benefits (Case 2) in Supply Chain Design (SCD) are compared. The results demonstrated that the first case has less computational complexity and better scalability, while the second case has more transparency, less congestion, and more security. As one of the main implications, supply chain managers who are focused on cost minimization as well as transparency maximization are advised to take into account the trade-off between featuring costs and benefits of blockchain technology.

## Introduction

Supply Chain Design (SCD) is mainly focused on cost, time, environment, and distance criteria, and efficiency criterion is less considered^1–4^. However, there are few articles in the literature that addressed efficiency along with other criteria. Grigoroudis et al.^5^ configured a biomass supply chain based on cost and efficiency criteria which was then developed by Petridis et al.^6^ developed by considering more criteria to calculate the efficiency. In this regard, Moheb-Alizadeh et al.^7^ discussed the efficiency of SCD in addition to cost and environmental issues. Generally, the criteria taken into account in the efficiency are related to service and cost and do not pay attention to transparency.

Transparency has become an important issue in supply chain planning with pressure from stakeholders^8^. For example, stakeholders may have concerns about practices and processes related to raw materials in the supply chain. By applying transparency in the supply chain, operations and products become clear for the stakeholders and such concerns are resolved. Blockchain can bring such transparency even to the entire supply chain^9^. In this regard, supply chain data is stored and recorded in blocks that cannot be manipulated^10^. In this way, the transparency expected by the stakeholders in the supply chain emerges. Therefore, blockchain technology can provide transparency in the supply chain. On the flip side, Transparency is one of the prominent characteristics of using blockchain, which leads to an increase in the level of trust, information integrity and visibility in the supply chain^11^. For this reason, by implementing blockchain in the supply chain from the transparency perspective, firms can build trust and gain better visibility of the supply chain. In a survey of supply chain leaders, it was reported that 40% of them tend to invest in blockchain technologies and 46% of leaders plan to use the Internet of Things (IoT)^12^. Forecasts show that by 2025, revenues from the application of blockchain will grow to 39 billion US dollars^13^. In a survey among 1280 respondents, 45% stated that they employ blockchain technology as a platform for information exchange in their companies^14^. The market size of next-generation supply chains, in which services are based on the digital revolution, was equal to 32 billion US dollars in 2019, and this number is expected to double by 2030^15^. The global distributed ledger market, which includes immutable records, smart contracts, digital identity, and proof-of-work, is dominated by supply chain audits and will increase to more than $103 billion by 2030^16^. With an annual growth rate of 53.2%, the blockchain supply chain market size is expected to reach $3272 million by 2026 from $253 million in 2020^17^.

Blockchain technology can share digital events among all blockchain members because it is a distributed database^18^. The data stored in each block is encrypted based on a value called “hash”^19^. In a blockchain, each block comprises the hash value of the previous block. Therefore, it becomes very difficult for an attacker to manipulate data on the blockchain^20,21^. One of the effects of using blockchain is transparency because blockchain leads to data immutability. In this regard, as much as the number of blocks increases, the level of transparency can increase^10^. The physical flow of the supply chain can be converted into a digital flow through IoT tools (such as QR code, Near-Field Communication (NFC), Radio-Frequency IDentification (RFID), online certification, etc.) and then converted to the blockchain network so that the stored information becomes immutable and the supply chain partners are able to confirm the information^22,23^. It is worth noting that, despite the many efforts of researchers, how blockchain is applied in the supply chain is still open to interpretation^24^.

In the following, the research works that have addressed the application of blockchain in the supply chain are reviewed under three topics of blockchain adoption in the supply chain, implementation of smart contracts through blockchain, and measuring transparency. Zheng et al.^25^ examined the adoption of blockchain in the spacecraft supply chain, which includes the spacecraft builder, supplier, and logistics service integrator under risk decision-making. The results showed that the overall profits of the supply chain grow with the blockchain adoption due to the two reasons of sharing information and reducing the cost of transactions. Xia et al.^26^ implemented fleet sharing through blockchain technology in the last mile delivery problem. Their results revealed that the higher the sharing power, the lower the costs. Munim et al.^27^ evaluated blockchain adoption strategies in the oil and gas industry through a decision-making method called Bayesian Best Worst Method (BWM). They found that expertise related to technology, collaboration, and operational costs had the greatest impact on blockchain adoption. Lack of expertise related to technology as well as lack of supply chain partner collaboration are barriers and decreasing operating costs is the driver of blockchain adoption. Zhang et al.^28^ dealt with the strategic pricing of two retailers (initial retailer and emerging retailer) in a competitive environment where they are able to decide whether to apply blockchain technology or not. They found that although information transparency leads to increased consumer desire, privacy data leakage hinders blockchain adoption. Therefore, the trade-off between the two can determine whether retailers use blockchain technology or not.

Zhang and Song^29^ investigated sustainability risk factors in blockchain adoption in the supply chain. It was proved that the increased costs and additional audits are among the most important risk factors for using this technology in a sustainable supply chain. Prajapati et al.^30^ integrated the normal and virtual closed-loop supply chain with regard to the IoT and Blockchain technology. For this purpose, a Mixed Integer Non-Linear Programming (MINLP) model was offered, which took into account energy consumption costs, tag purchase costs, security costs, and data non-manipulation costs in addition to the common costs of the supply chain. Zeng et al.^31^ established information sharing between suppliers, manufacturers and distributors based on blockchain technology. Applying blockchain can lead to the identification of less efficient nodes in the supply chain scheduling problem. De Carvalho et al.^32^ analyzed the deployment of blockchain in SCD where the information of the transportation time is stored in the blockchain. They found that the partial adoption of the blockchain in the supply chain can improve the total profit value.

Rahmanzadeh et al.^33^ made the design part of the supply chain dependent on external capabilities. To protect the intellectual property of those who present their innovative design, the authors used blockchain technology. After registering the idea by the designer and producing the products in the product chain, the designers receive their reward. The obtained results demonstrated that considering blockchain in tactical supply chain planning can reduce the costs of non-original designs. Dolgui et al.^34^ presented a smart contract in the form of flexible flow shop scheduling on the blockchain platform so that in this contract logistics companies are assigned to jobs and their operations are scheduled. The companies participate in the design of the blockchain through the information service resulting from the executing operation. Manupati et al.^35^ implemented a smart contract through blockchain technology in order to monitor supply chain performance to optimize operating costs and emission levels in the production allocation problem. They found that the blockchain approach was successful in reducing costs and emission levels. Wang et al.^36^ addressed information sharing between upstream and downstream members of the supply chain through blockchain, where the supplier adjusts its inventory level based on the retailers' demand. For this purpose, they designed a blockchain system that focuses on data usage tracking, proper data valuation, and fair compensation.

Bai and Sarkis^37^ evaluated blockchain technologies to be employed in the supply chain through a decision-making approach based on fuzzy logic. In addition to technical characteristics, transparency factors for sustainability were among the criteria used in this evaluation. Maity et al.^10^ applied blockchain to the sausage supply chain. By creating a relationship between supply chain nodes and produced blocks, they introduced a measure called transparency. By increasing the number of blocks, more transparency is provided in the supply chain, and such an increase in transparency makes it hard for the attacker to manipulate the supply chain data.

Table 1 provides a comparative analysis of the most relevant research works in summary.

**Table 1:** Comparative analysis of the relevant studies.

References	Network scale	Efficient network design	Objective function(s)	IoT costs and benefits	Blockchain technology			Blockchain with stochastic conditions	Solution approach
					Blockchain adoption	Smart contract	Transparency		
Grigoroudis et al.^5^	Two-level	✓	Cost minimization						Branch and efficiency (B&E) algorithm
Petridis et al.^6^	Two-level	✓	Cost minimization						B&E algorithm
Rahmanzadeh et al.^33^	Three-level		Profit maximization			✓			Fuzzy set theory
Dolgi et al. (2020)	Two-level		Trade-off between SC lead-time and contract costs			✓			Optimal control and mathematical programming
Manupati et al.^35^	Three-level		Minimizing costs and emission levels			✓			MINLP model
Bai and Sarkis^37^	–		Selecting the most suitable blockchain technology				✓		Group decision method
Wang et al.^36^	Two-level		Cost minimization			✓			Monte Carlo Simulation
Moheb-Alizadeh et al.^7^	Four-level	✓	Profit maximization; Emission minimization; efficiency maximization; social responsibility maximization						Lagrangian relaxation
Zheng et al.^25^	Three-level		Profit maximization		✓				Stackelberg game
Xia et al.^26^	Two-level		Cost minimization		✓				Branch and price algorithm
Maity et al.^10^	Five-level		Batch dispersion minimization				✓		L-shaped method
Munim et al.^27^	–		Determining the most preferred strategy for the adoption of blockchain		✓				BWM method
Zhang et al.^28^	Single-level		Profit maximization		✓				–
Zhang and Song^29^	–		Determining risk factors in blockchain adoption		✓				Failure mode and effect analysis and BWM method
Prajapati et al.^30^	Five-level		Total expected revenue maximization	✓	✓				MINLP model
Cai et al.^49^	Three-level		Cost minimization		✓				Sequential brain storm optimization algorithm
De Carvalho et al.^32^	Two-level		Profit maximization	✓	✓				Mixed-integer quadratic programming model
This work	Two-level	✓	Cost minimization; Transparency maximization	✓			✓	✓	Improved B&E algorithm, CCP, & FGP

As can be seen in Table 1, the previous studies ignored the simultaneous consideration of cost objective functions (related to SCD and blockchain) and transparency (resulting from the blockchain network). In addition, in the design of the blockchain network, stochastic conditions are not investigated. It is noteworthy that nowadays, there is a need for the stakeholders of a supply chain to have a common understanding and access to information about the product without delay and without distortion^38^. To fulfill this necessity, it is essential to make supply chains transparent. Blockchain technology can deploy such transparency in supply chains^37^. Therefore, it is an important motivation for this work to integrate supply chain planning and blockchain technology. In this regard, this work integrates supply chain network design and blockchain under stochastic conditions to provide a practical decision support system. Therefore, the main contributions of the study are listed below:

i. Integrating supply chain network design with blockchain technology,
ii. Designing the supply chain based on transparency criterion in addition to common criteria such as cost and service,
iii. Partial design of the blockchain network and supply chain with regard to efficiency criterion,
iv. Considering stochastic conditions for blockchain to be modeled using Chance-Constrained Programming (CCP),
v. Presenting a bi-objective mathematical model based on cost and transparency and treating it based on Fuzzy Goal Programming (FGP),
vi. Providing different analyses on cost, transparency, and service based on the simultaneous design of blockchain and supply chain,
vii. Developing an improved B&E algorithm according to the proposed FGP and blockchain adoption criterion.

The rest of the manuscript is structured as follows. “Problem descriptions and models” section describes the problem as well as the developed bi-objective optimization model. The proposed methodology is elaborated in “Methododlogy” section. “Numerical results and analysis” section represents the obtained numerical results along with practical implications. Finally, “Conclusion and outlook” section gives the concluding remarks and draws the outlook of the research.

## Problem descriptions and models

Here, the aim is to integrate blockchain technology with SCD to configure a blockchain-enabled three-level supply chain network. The examined supply chain includes three echelons (factory, warehouse and customer). Warehouses are the members of the supply chain that can be decided about their installation or non-installation, but other members are already available. The products are produced in the plant and shipped from the first layer to the second layer of the supply chain. Then, products are shipped from warehouses to demand areas to meet customer demand. Here, costs are related to the transportation and installation activities. The decision variables include the installation of warehouses, the installation of links between two levels, and the amount of goods in the flow at each stage of the supply chain. There are two objective functions in this work for supply chain planning. One of the two is related to the minimization of the mentioned costs. Another objective function is transparency. Members of the second layer of the supply chain can be equipped with IoT tools to convert the physical flow of the supply chain into a digital flow and thus generate blocks. To form a chain of blocks, it is necessary that at least one member of the first layer and also one member of the second layer are connected to the equipped member of the second layer.

Since the blockchain concept is based on decentralization, the more blocks produced by the equipped warehouses, the greater the transparency. It is assumed the transparency criterion is based on the probability that the attacker will not succeed in manipulating the blockchain. Furthermore, the distribution of such probability is “negative binomial (According to Maity et al.^10^, ${\mathrm{P}}_{\mathrm{failure}}=(\frac{r+k-1}{k}){{\mathrm{P}}_{\mathrm{H}}}^{r}{{\mathrm{P}}_{\mathrm{a}}}^{k}{(1-\frac{{\mathrm{P}}_{\mathrm{a}}}{{\mathrm{P}}_{\mathrm{H}}})}^{k}$ determines the probability that the attacker will fail (${\mathrm{P}}_{\mathrm{H}}$, ${\mathrm{P}}_{\mathrm{a}}$ and $k$ indicate probability related to an honest node, probability related to an attacker, and the number of failures, respectively). There is an assumption wherein $r$ is equal to 1; i.e., the first time the attacker can manipulate the data, he/she can take control of the entire blockchain. For more details, please see Page 9 in Maity et al.^10^.)”. The reason for choosing this distribution by Maity et al.^10^ is that compared to other distributions, negative binomial distribution has the ability to consider the number of failures and successes of attackers, where the attacker intends to manipulate the supply chain data recorded in blocks. Each member of the second layer of the supply chain incurs the costs of being equipped with IoT tools in order to create blockchain infrastructure and provide services to other levels. In this study, this cost is regarded as a proportion of the installation costs of that member. Members who join the blockchain will benefit from advantages such as transparency, tracking, better planning and security. These benefits can lead to cost savings. Part of these savings can be taken into account as revenue in the supply chain. Considering that the members of the blockchain must be negotiated to know the benefits, and these benefits are interactive between the members, these benefits are treated as a factor of the cost of interactions between levels (such as the cost of transportation). SCD and blockchain are integrated into Eqs. (1)–(27). The notation related to the equations is given in Table 2.

**Table 2:** Indices, parameters and decision variables.

	Descriptions
Indices	
$i\in I$	Index of plants
$j\in J$	Index of warehouses
$k\in K$	Index of customers
$t\in T$	Index of iterations
$q\in Q$	Index of inputs used at each Decision-Making Unit (DMU)
$p\in P$	Index of outputs produced at each DMU
$b\in B$	Index of blocks generated by blockchain technology in the supply chain
Parameters	
${p}_{i}^{u}$	Upper bound of produced quantities at plant *i*
${p}_{i}^{l}$	Lower bound of produced quantities at plant *i*
${q}_{ij}^{u}$	Upper bound of transported quantities from plant *i* to warehouse *j*
${q}_{jk}^{u}$	Upper bound of transported quantities from warehouse *j* to customer *k*
${w}_{j}^{u}$	Upper capacity of warehouse *j*
${\beta }_{j}$	Coefficient relating quantity at capacity at warehouse *j*
${I}_{j}^{0}$	Inventory level stored at warehouse *j*
${c}_{i}^{p}$	Production cost at plant *i*
${c}_{ij}^{v}$	Unit transportation cost of products transported from plant *i* to warehouse *j*
${c}_{ij}^{f}$	Route transportation cost of products transported from plant *i* to warehouse *j*
${c}_{jk}^{v}$	Unit transportation cost of products transported from warehouse *j* to customer *k*
${c}_{jk}^{f}$	Route transportation cost of products transported from warehouse *j* to customer *k*
${f}_{j}^{c}$	Installation cost of warehouse *j*
${d}_{k}^{R}$	Demand of customer *k*
$\upeta$	Level of service
${f}_{b}^{pr}$	Measure of progress made by the attacker in terms of its failure based on the number of blocks type *b* generated by independent warehouses
${B}_{j}^{A}$	Blockchain technology adoption parameter in warehouse *j* in order to create transparency in the supply chain
$\underline{B}, \overline{B }$	Minimum and maximum level of transparency expected by the supply chain manager
${B}^{N}$	Minimum number of warehouses participating in the blockchain
$\rho$	Conversion factor of the installation cost to the cost of using the blockchain
$\gamma$	Conversion factor of the variable transportation cost to the cost of using the blockchain
${a}_{j}$	Solutions with efficiency score greater than or equal to the threshold of the supply chain manager that are selected with ${\upxi }_{j}$
${I}_{jq}^{D}$	Amount of input *q* for DMU *j*
${O}_{jp}^{D}$	Amount of output *q* for DMU *j*
$\varepsilon$	Non*-*Archimedean *infinitesimal* epsilon
$E\left(\widetilde{\xi }\right), Var\left(\widetilde{\xi }\right)$	Expected value and variance of random parameter
$1-{\alpha }^{s}$	Confidence level for chance constraint
${Z}_{1-{\alpha }^{s}}$	Inverse function of the standard normal cumulative distribution function
${f}^{I,tr},{f}^{I,co}$	Aspiration levels (ideal solutions)
${f}^{N,tr},{f}^{N,co}$	Aspiration levels (nadir solutions)
${\theta }^{tr},{\theta }^{co}$	Weight associated with each fuzzy goal
Decision variables	
${p}_{i}$	Production quantity at plant *i*
${q}_{ij}^{1\to 2}$	Transported quantity from plant *i* to warehouse *j*
${q}_{jk}^{2\to 3}$	Transported quantity from warehouse *j* to customer *k*
${w}_{j}$	Capacity of warehouse *j*
${g}_{k}$	Percentage of unmet demand of customer k
${x}_{ij}^{1\to 2}$	1 if the connection between plant *i* and warehouse *j* exists, 0 otherwise
${x}_{jk}^{2\to 3}$	1 if the connection between warehouse *j* and customer *k* exists, 0 otherwise
${y}_{j}$	1 if warehouse *j* will be installed, 0 otherwise
${B}_{b}^{T}$	Total number of blocks type *b* (related to the second layer of the supply chain that are extracted from independent warehouses
${B}_{j}^{DN}$	1 if warehouse *j* is equipped with an IoT tool to produce the block, 0 otherwise
${B}_{ij}^{1\to 2}$	1 if warehouse *j* and plant *i* participate to form the blockchain, 0 otherwise
${B}_{jk}^{2\to 3}$	1 if warehouse *j* and customer *k* participate to form the blockchain, 0 otherwise
${\upxi }_{j}$	1 if warehouse *j* will be installed under efficiency level *a* %, 0 otherwise
${\vartheta }_{jq}$	Weight assigned to input *q* for DMU *j*
${\mu }_{jp}$	Weight assigned to output *p* for DMU *j*
${d}_{j}$	Level of inefficiency of DMU *j*
${\omega }_{j}$	Level of efficiency of DMU $j$
${F}^{tr},{F}^{co}$	Objective functions related to transparency maximization and costs minimization

Now, the developed model is given as follows:

1 $${ }\begin{array}{*{20}c} {{\text{maximize}}} &\; {F^{tr} = \mathop \sum \limits_{b \in B} f_{b}^{pr} B_{b}^{T} ,} \\ \end{array}$$

2 $$\begin{aligned} {{\text{minimize}}} {F}^{co} & = \mathop \sum \limits_{j \in J} \rho f_{j}^{c} B_{j}^{DN} - \gamma (\mathop \sum \limits_{i \in I} \mathop \sum \limits_{j \in J} c_{ij}^{v} B_{ij}^{1 \to 2} + \mathop \sum \limits_{j \in J} \mathop \sum \limits_{k} c_{jk}^{v} B_{jk}^{2 \to 3} ) + \mathop \sum \limits_{i \in I} c_{i}^{p} p_{i} + \mathop \sum \limits_{i \in I} \mathop \sum \limits_{j \in J} c_{ij}^{v} q_{ij}^{1 \to 2} \\ &\quad+ \mathop \sum \limits_{i \in I} \mathop \sum \limits_{j \in J} c_{ij}^{f} x_{ij}^{1 \to 2} + \mathop \sum \limits_{j \in J} \mathop \sum \limits_{k \in K} c_{jk}^{v} q_{jk}^{2 \to 3} + \mathop \sum \limits_{j \in J} \mathop \sum \limits_{k \in K} c_{jk}^{f} x_{jk}^{2 \to 3} + \mathop \sum \limits_{j \in J} f_{j}^{c} y_{j} , \end{aligned}$$

3 $${ }\begin{array}{*{20}c} {\text{subject to}} & {p_{i} = \mathop \sum \limits_{j \in J} q_{ij}^{1 \to 2} } & \quad {\forall i \in I} \\ \end{array} ,$$

4 $$\begin{array}{*{20}c} {p_{i} \le p_{i}^{u} } & \quad {\forall i \in I} \\ \end{array} ,$$

5 $$\begin{array}{*{20}c} {p_{i}^{l} \le p_{i} } & \quad {\forall i \in I} \\ \end{array} ,$$

6 $$\begin{array}{*{20}c} {\mathop \sum \limits_{i \in I} q_{ij}^{1 \to 2} = \mathop \sum \limits_{k \in K} q_{jk}^{2 \to 3} } & \quad {\forall j \in J} \\ \end{array} ,$$

7 $$\begin{array}{*{20}c} {q_{ij}^{1 \to 2} \le q_{ij}^{u} x_{ij}^{1 \to 2} } & \quad {\forall i \in I;j \in J} \\ \end{array} ,$$

8 $$\begin{array}{*{20}c} {q_{jk}^{2 \to 3} \le q_{jk}^{u} x_{jk}^{2 \to 3} } & \quad {\forall j \in J;k \in K} \\ \end{array} ,$$

9 $$\begin{array}{*{20}c} {q_{ij}^{1 \to 2} \le y_{j} } & \quad {\forall i \in I;j \in J} \\ \end{array} ,$$

10 $$\begin{array}{*{20}c} {q_{jk}^{2 \to 3} \le y_{j} } & \quad {\forall j \in J;k \in K} \\ \end{array} ,$$

11 $$\begin{array}{*{20}c} {w_{j} \ge \beta_{j} \left( {\mathop \sum \limits_{i \in I} q_{ij}^{1 \to 2} + I_{j}^{0} } \right)} & \quad {\forall j \in J} \\ \end{array} ,$$

12 $$\begin{array}{*{20}c} {w_{j} \le w_{j}^{u} y_{j} } & \quad {\forall j \in J} \\ \end{array} ,$$

13 $$\begin{array}{*{20}c} {g_{k} = d_{k}^{R} - \mathop \sum \limits_{i} q_{jk}^{2 \to 3} } & \quad {\forall k \in K,} \\ \end{array}$$

14 $$\mathop \sum \limits_{k \in K} g_{k} \le \left( {1 - {\upeta }} \right)\mathop \sum \limits_{k \in K} d_{k}^{R} ,$$

15 $$\begin{array}{*{20}c} {B_{j}^{DN} \le y_{j} } & \quad {\forall j \in J} \\ \end{array} ,$$

16 $$\begin{array}{*{20}c} {B_{j}^{DN} \le \mathop \sum \limits_{i} B_{ij}^{1 \to 2} } & \quad {\forall j \in J} \\ \end{array} ,$$

17 $$\begin{array}{*{20}c} {B_{j}^{DN} \le \mathop \sum \limits_{k} B_{jk}^{2 \to 3} } & \quad {\forall j \in J} \\ \end{array} ,$$

18 $$\begin{array}{*{20}c} {B_{ij}^{1 \to 2} \le x_{ij}^{1 \to 2} } & \quad {\forall i \in I;j \in J} \\ \end{array} ,$$

19 $$\begin{array}{*{20}c} {B_{jk}^{2 \to 3} \le x_{jk}^{2 \to 3} } & \quad {\forall j \in J;k \in K} \\ \end{array} ,$$

20 $$\begin{array}{*{20}c} {B_{ij}^{1 \to 2} \le B_{j}^{DN} } & \quad {\forall i \in I;j \in J} \\ \end{array} ,$$

21 $$\begin{array}{*{20}c} {B_{ij}^{1 \to 2} \le B_{j}^{DN} } & \quad {\forall i \in I;j \in J} \\ \end{array} ,$$

22 $$\underline {B} \le \mathop \sum \limits_{j \in J} B_{j}^{A} B_{j}^{DN} \le \overline{B},$$

23 $$\mathop \sum \limits_{j \in J} B_{j}^{DN} \ge B^{N} ,$$

24 $$\mathop \sum \limits_{j \in J} B_{j}^{DN} = \mathop \sum \limits_{b \in B} \left| b \right|B_{b}^{T} ,$$

25 $$\mathop \sum \limits_{b \in B} B_{b}^{T} = 1,$$

26 $$\begin{array}{*{20}c} {p_{i} , q_{ij}^{1 \to 2} , q_{jk}^{2 \to 3} , w_{j} , g_{k} \ge 0} & \quad {\forall i \in I;j \in J;k \in K} \\ \end{array} ,$$

27 $$\begin{array}{*{20}c} {x_{ij}^{1 \to 2} , x_{jk}^{2 \to 3} , y_{j} ,B_{ij}^{1 \to 2} ,B_{jk}^{2 \to 3} ,B_{b}^{T} ,B_{j}^{DN} \in \left\{ {0,1} \right\}} & \quad {\forall i \in I;j \in J;k \in K;b \in B.} \\ \end{array}$$

The first objective function maximizes the total number of blocks extracted from the second layer of the supply chain. Due to this function, the network aims to be decentralized. On the other hand, where an attacker intends to manipulate data by attacking the blockchain, the attacker’s progress (in terms of failure); i.e., parameter ${f}_{b}^{pr}$ (when attacking the chain), is obtained by summing the negative binomial distribution at each step of the attack. The greater the number of blocks ($b$), the higher the failure progress because more distributions related to the number of blocks are added together. Therefore, the attacker’s success decreases drastically as $b$ increases. The second objective function seeks to minimize the costs such as the fixed and variable costs of transportation in each stage of the supply chain, production costs, and warehouse installation costs. The benefits of supply chain members from joining the blockchain and the cost of using the blockchain are also shown in the second objective function. Constraint (3) balances the number of produced goods in the first layer of the supply chain and the number of products received by warehouses in the second layer of the supply chain. Constraints (5) and (6) state the upper and lower thresholds of production in each plant. Constraint (6) balances the number of products that are received by the warehouse from the first layer of the supply chain and the number of products that are shipped from the warehouse to the customers in the third level of the supply chain. Constraints (7) and (8) guarantee that if and only if there is a connection between two levels of the supply chain, the products of each stage are shipped according to the capacity of the connection. Constraints (9) and (10) create a relationship between the installation of warehouses and their connections. Constraints (11) and (12) determine the minimum and maximum capacity of each warehouse. Constraint (11) is based on the coefficient of the number of products received by the warehouse and the initial inventory of the warehouse. Constraint (12) is on the basis of the upper capacity of each warehouse. Constraint (13) specifies the amount of unmet demand for each customer. Constraint (14) indicates the upper threshold of the unmet demand for all customers. Constraints (15)–(25) show the relationships between supply chain network and blockchain. Constraint (15) expresses that the block can be produced if the warehouse is installed. Constraints (16) and (17) guarantee that for the presence of warehouse *j* in the blockchain, at least one member of the first layer (plants) and at least one member of the third level (customers) of the supply chain are present in that chain. Constraints (18) and (19) explain that the blockchain is formed when there is a physical flow between the levels of the supply chain. Constraints (20) and (21) indicate that the blockchain is formed when warehouse *j* is equipped with an IoT tool for block production. Constraint (22) determines the transparency thresholds expected of the supply chain manager. With the aim of decentralization, the minimum number of independent warehouses in the blockchain is guaranteed in constraint (23). Constraints (24) and (25) specify the total number of independent blocks produced in the second layer. Constraints (26) and (27) show non-negative continuous and binary decision variables, respectively.

## Methodology

### Improved branch and efficiency algorithm

**Figure 1:**
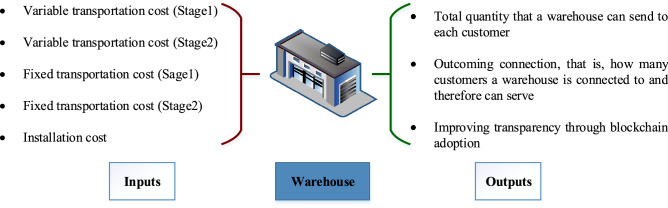
Solution evaluation criteria based on cost, service, and transparency.

As discussed before, most studies focus on the impacts of cost, time, service, and other criteria and ignore the efficiency criterion. In this research, the efficiency of the solutions obtained from the optimization model is computed through Data Envelopment Analysis (DEA). In this way, not only the solutions are optimal but also efficient. For this purpose, an improved B&E algorithm is developed and applied to take into account transparency as well as service and cost. In this regard, Eqs. (1)–(27) are called “Master Problem”. After solving the master problem, installed warehouses (solutions) are determined. Unlike other research works, in addition to cost and service, the solutions are evaluated based on transparency criteria as shown in Fig. 1.

To evaluate the solutions, the Simultaneous Data Envelopment Analysis (SDEA) is utilized. For more information, please see Klimberg and Ratick^39^. SDEA takes into account transparency (called SDEAT) in addition to common criteria such as cost and service according to Fig. 1. In the SDEAT model, the index $j$ represents decision-making units (which denotes warehouses in our proposed mathematical model), $p$ represents outputs (based on transparency and service criteria), and $q$ represents inputs (types of costs). The objective function, Eq. (28), maximizes the level of efficiency of DMUs. Constraint (29) measures the efficiency of each DMU. The weighted sum of inputs is optionally set equal to 1 in Constraint (30). Constraint (31) calculates the efficiency of each DMU according to the weighted sum of outputs. Constraint (32) sets the upper threshold of the efficiency of each DMU equal to 1. Constraints (33) and (34) indicate positive continuous and non-negative continuous decision variables, respectively.

28 $$\begin{array}{*{20}c} {{\text{maximize}}} & {\mathop \sum \limits_{j \in J} \omega_{j} } \\ \end{array} ,$$

29 $$\begin{array}{*{20}c} {\text{subject to}} & {\omega_{j} = 1 - d_{j} } & \quad {\forall j \in J,} \\ \end{array}$$

30 $$\begin{array}{*{20}c} {\mathop \sum \limits_{q \in Q} \vartheta_{jq} I_{jq}^{D} = 1} &\quad {\forall j \in J,} \\ \end{array}$$

31 $$\begin{array}{*{20}c} {\mathop \sum \limits_{p \in P} \mu_{jp} O_{jp}^{D} + d_{j} = 1} &\quad {\forall j \in J,} \\ \end{array}$$

32 $$\begin{array}{*{20}c} {\mathop \sum \limits_{p \in P} \mu_{jp} O_{lp}^{D} - \mathop \sum \limits_{q \in Q} \vartheta_{jq} I_{lq}^{D} \le 0} & \quad{\forall j \in J;l \in L; l \ne j,} \\ \end{array}$$

33 $$\begin{array}{*{20}c} {\mu_{jp} , \vartheta_{jq} \ge \varepsilon } &\quad {\forall j \in J;p \in P;q \in Q,} \\ \end{array}$$

34 $$\begin{array}{*{20}c} {d_{j} , \omega_{j} \ge 0} &\quad {\forall j \in J.} \\ \end{array}$$

When the solutions of the master problem are evaluated by Eqs. (28)–(34) based on transparency, cost, and service criteria, the most efficient solutions are determined. Efficient solutions are the solutions whose efficiency is greater than the minimum efficiency expected by supply chain managers. Equation (35) shows the efficient solutions.

35 $${\upxi }_{j} = \begin{array}{*{20}c} {\left\{ {\begin{array}{*{20}c} {1,} & {\omega_{j} \ge a_{j} ,} \\ {0,} & {{\text{otherwise}},} \\ \end{array} } \right.} &\quad {\forall j \in J} \\ \end{array} .$$

Based on the efficient solutions, the master problem should be updated. For this purpose, efficient solutions are added to the master problem as feasible solutions through “efficiency cuts”. The constraints related to efficiency cuts are shown in Eqs. (36)–(41). Therefore, all the terms involved in the master problem with the warehouse installation are updated by Eqs. (36)–(41):

36 $$\begin{array}{*{20}c} {{\text{minimize}}} & {\mathop \sum \limits_{j \in J} f_{j}^{c} {\upxi }_{j} } \\ \end{array} ,$$

37 $$\begin{array}{*{20}c} {\text{subject to}} & {q_{ij}^{1 \to 2} \le {\upxi }_{j} } &\quad {\forall i \in I;j \in J} \\ \end{array} ,$$

38 $$\begin{array}{*{20}c} {q_{jk}^{2 \to 3} \le {\upxi }_{j} } &\quad {\forall k \in K;j \in J} \\ \end{array} ,$$

39 $$\begin{array}{*{20}c} {w_{j} \ge b_{j} \left( {\mathop \sum \limits_{i \in I} q_{ij}^{1 \to 2} + {\upxi }_{j} i_{j}^{0} } \right)} &\quad {\forall j \in J,} \\ \end{array}$$

40 $$\begin{array}{*{20}c} {w_{j} \le w_{j}^{u} {\upxi }_{j} } &\quad {\forall j \in J,} \\ \end{array}$$

41 $$\begin{array}{*{20}c} {B_{j}^{DN} \le {\upxi }_{j} } &\quad {\forall j \in J.} \\ \end{array}$$

**Figure 2:**
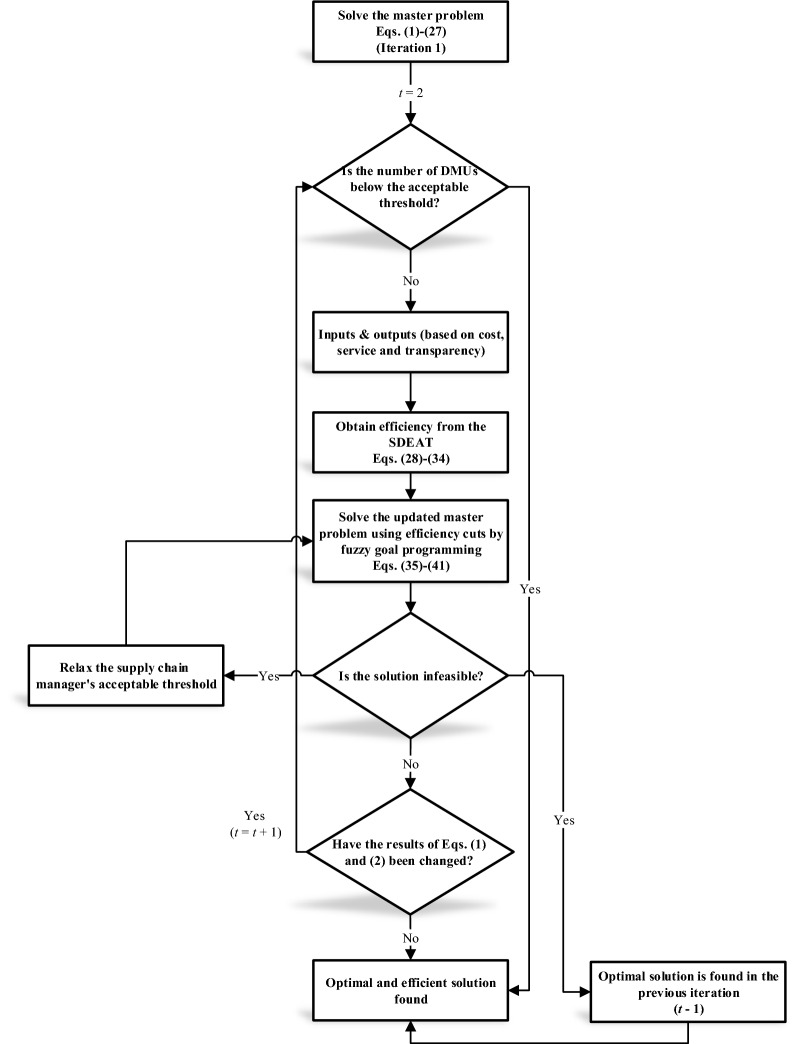
Flowchart of the proposed B&E algorithm.

The master problem, which is updated with efficiency cuts, can be re-solved. The results extracted from the updated master problem are evaluated by criteria based on cost, service, and transparency. Once more, the master problem can be updated by efficiency cuts. As can be seen, such a solution process is iterative to find both optimal and efficient solutions. For this reason, the B&E algorithm is implemented to treat the proposed model in this research, which is shown in Fig. 2.

In the first iteration of the algorithm, the master problem is solved. It is necessary to check the number of DMUs (warehouses) because the DEA model does not have sufficient ability to evaluate very small numbers of DMUs. In addition, the supply chain manager may set a minimum number of supply chain members. Then, based on the solutions extracted from the master problem, the values of the criteria introduced in Fig. 1 are determined.

The solutions are evaluated by the SDEAT model and the most efficient solution is specified according to the threshold requested by the supply chain manager. Efficient solutions are added to the master problem by efficiency cuts. The updated master problem is solved. If the problem space is infeasible, then the supply chain manager should either accept the solution of the previous iteration as the optimal solution or reduce his/her acceptance threshold in terms of efficiency so that more solutions can be considered in the updated master problem. If the updated master problem is not infeasible, then the values of the objective functions are checked. If these values do not change, the algorithm terminates. Otherwise, the algorithm is implemented on the updated master problem. Various conditions can be taken into account for the termination of this algorithm. Achieving a certain number of solutions, running a certain number of iterations, no change in the values of the objective functions in two consecutive iterations, etc. are among these conditions.

#### Proposition

If ${S}^{f,1}$ represents the solution space of the master problem, Eqs. (1)–(26), and ${S}^{f,t}$ denote the solution spaces of the subsequent iterations which contain efficiency cuts, then we have ${F}^{co,1}\ge {F}^{co,2}\ge \dots \ge {F}^{co,T}$, where ${F}^{co,t}(t=\mathrm{0,1},\dots ,T)$ stands for the cost objective function in each iteration.

#### Proof

In each iteration, a set of constraints called efficiency cuts is added to the optimization model of the previous iteration. In this way, the number of DMUs is reduced because inefficient DMUs are eliminated. As a result, continuous and binary decision variables related to inefficient DMUs are removed from the optimization model. For this reason, in each iteration, the feasible space is reduced. Therefore, in each iteration, the value of the cost objective function decreases.

### Uncertainty treatment: chance-constraint programming

Nowadays, blockchain has attracted a lot of attention from industries and research due to its many advantages (including security, traceability, and transparency), and this has made blockchain be used in a wide range of applications, including supply chain management. However, the large geographical spread of some supply chains, generation, and transfer of blocks are among the things that increase the possibility of insecurity in the blockchain. Therefore, considering stochastic conditions in the blockchain is recommended by researchers^40,41^. Furthermore, since blockchain is applied in supply chain management, it is clear that real-world conditions affect it where there is no real-world certainty. CCP is one of the methods of optimization programming and one of the types of stochastic programming that can investigate random data variations^42^.

CCP can be converted into deterministic equivalents^43^. One of the famous distributions used for the randomness of parameters in this programming is the normal distribution^44^. In this research, it is supposed that ${B}^{N}$ is under uncertain conditions and this parameter follows a normal distribution. Therefore, uncertainty is regarded where it affects supply chain members and blockchain. Equation (42) indicates the stochastic parameter related to the minimum number of blocks in the blockchain. Equation (43) expresses the chance constraint. Equation (44) reformulates the chance constraint based on the standard normal distribution. Equation (45) displays the cumulative distribution function. According to Eq. (45), the chance constraint is re-written in Eq. (46). Equation (47) specifies the inverse cumulative distribution function. The deterministic equivalent of chance constraint is given in Eq. (48), which is a simple form of the previous equation.

42 $$\widetilde{{\varvec{\xi}}}={\widetilde{B}}^{N},$$

43 $$P\left(\sum_{j}{B}_{j}^{DN}\ge \widetilde{\xi }\right)\ge 1-{\alpha }^{s},$$

44 $$P\left(\sum_{j}{B}_{j}^{DN}\ge \widetilde{\xi }\right)=P\left(\frac{\widetilde{\xi }-E(\widetilde{\xi })}{\sqrt{Var(\widetilde{\xi })}}\le \frac{\sum_{j}{B}_{j}^{DN}-E(\widetilde{\xi })}{\sqrt{Var(\widetilde{\xi })}}\right),$$

45 $$P\left(\frac{\xi -E(\xi )}{\sqrt{Var(\xi )}}\le \frac{\sum_{j}{B}_{j}^{DN}-E(\widetilde{\xi })}{\sqrt{Var(\widetilde{\xi })}}\right)=F\left(\frac{\sum_{j}{B}_{j}^{DN}-E(\widetilde{\xi })}{\sqrt{Var(\widetilde{\xi })}}\right),$$

46 $$F\left(\frac{\sum_{j}{B}_{j}^{DN}-E(\widetilde{\xi })}{\sqrt{Var(\widetilde{\xi })}}\right)\ge 1-{\alpha }^{s},$$

47 $$\frac{\sum_{j}{B}_{j}^{DN}-E(\widetilde{\xi })}{\sqrt{Var(\widetilde{\xi })}}\ge {Z}_{1-{\alpha }^{s}},$$

48 $$\sum_{j}{B}_{j}^{DN}\ge E\left(\widetilde{\xi }\right)+{Z}_{1-{\alpha }^{s}}\sqrt{Var\left(\widetilde{\xi }\right)}.$$

### Fuzzy goal programming

The master problem studied in this work has two objective functions. Generally, to solve multi-objective problems, it is necessary to transform the objective functions into a single objective function. One of the methods that transform multi-objective problems into single-objective problems is Goal Programming (GP)^45^. In this programming, an aspiration level (goal) is determined for each objective function. The new objective function is the minimization of the sum of the deviations of the objective functions from the goals. But determining a deterministic value for the aspiration levels is difficult for decision-makers and managers for various reasons such as uncertain conditions. For this reason, Zimmermann^46^ developed a model that takes ambiguous goals into account. On the other hand, Tiwari et al.^47^ offered a fuzzy programming model to incorporate ambiguity into the goals. They calculated the ideal and anti-ideal values of the objective functions and based on these values, they transformed the objective functions into membership functions. Finally, the objective functions were transformed into the maximization of the weighted sum of the membership functions. Here, the method suggested by Tiwari et al.^47^ is implemented to solve the bi-objective problem. The aspiration levels for Objective Functions (1) and (2) are defined in Eqs. (49) and (50) based on fuzzy goals. In these Equations, this sign “$\lesssim$” indicates the term “approximately less than or equal to”.

49 $${f}^{I,tr}\lesssim {F}^{tr},$$

50 $${F}^{co}\lesssim {f}^{I,co}.$$

To transform the objective functions into membership functions, it is necessary to calculate the ideal and anti-ideal values for the objective functions. Ideal values are obtained when each objective function is optimized alone. When one objective function is optimized alone, the anti-ideal value of the other objective function can be computed. Based on this, Objective Functions (1) and (2) become membership functions in Eqs. (51) and (52).

51 $${\mu }^{{F}^{tr}}=\left\{\begin{array}{l}1, \\ 1-\frac{{f}^{I,tr}-{F}^{tr}}{{f}^{N,tr}-{f}^{I,tr}}\\ 0, \end{array}\right., \begin{array}{l}{F}^{tr}\ge {f}^{I,tr}, \\ {f}^{I,tr}\le {F}^{tr}\le {f}^{N,tr},\\ {F}^{tr}\le {f}^{N,tr},\end{array}$$

52 $${\mu }^{{F}^{co}}=\left\{\begin{array}{l}1, \\ 1-\frac{{F}^{co}-{f}^{I,co}}{{f}^{N,co}-{f}^{I,co}},\\ 0, \end{array}\right.\begin{array}{l}{f}^{co}\le {F}^{I,co}, \\ { f}^{I,co}\le {F}^{co}\le {f}^{N,co}\\ {F}^{co}\ge {f}^{N,co}. \end{array}.$$

In order to transform the problem of the bi-objective optimization model into a single-objective optimization model, it is necessary to consider the weighted sum of the membership functions obtained in Eqs. (51) and (52) as the new objective function. Other Equations (i.e., Constraints (3)–(27)) do not change. Therefore, Eqs. (53)–(56) represent the single-objective optimization model, which is the single-objective counterpart of Eqs. (1)–(27).

53 $$\begin{array}{*{20}c} {{\text{maximize}}} & {\theta^{tr} \mu^{{F^{tr} }} + \theta^{co} \mu^{{F^{co} }} } \\ \end{array} ,$$

54 $$\begin{array}{*{20}c} {\text{Subject to}} & {{\text{Eqs}}. \, \left( {3} \right) - \left( {{27}} \right),} \\ \end{array}$$

55 $$\mu^{{F^{tr} }} ,\mu^{{F^{co} }} \le 1,$$

56 $$\mu^{{F^{tr} }} ,\mu^{{F^{co} }} \ge 0.$$

#### Theorem

Equations (53)–(56) generate a solution that is a Pareto efficient solution for the model presented in Eqs. (1)–(27).

#### Proof

Let Eqs. (53)–(56) generate the optimal solution “${\varphi }^{*}$”, where the optimal decision variables are indicated by ${\varphi }^{*}$. If ${\varphi }^{*}$ is not an efficient solution for Eqs. (1)–(27), then there is another feasible solution, such as ${\gamma }^{*}$, which can generate better objective function values compared to ${\varphi }^{*}$. In other words, ${\gamma }^{*}$ is a solution that is not worse than ${\varphi }^{*}$ and is better than ${\varphi }^{*}$ in at least one of the values of the objective functions. Therefore, the membership functions derived by ${\gamma }^{*}$ are not worse than the membership functions derived by ${\varphi }^{*}$, and at least one of the membership functions derived by ${\gamma }^{*}$ is better than ${\varphi }^{*}$. This means that ${\gamma }^{*}$ is the optimal solution of Eqs. (53)–(56), which is in contradiction with the optimality of ${\varphi }^{*}$.

## Numerical results and analysis

Here, the application and validation of the developed models within the given framework of the methodology are demonstrated based on the illustrative example in Petridis et al.^6^. The examined supply chain has two stages including plant-warehouse and warehouse-customer. In this supply chain, there are 5 plants, 20 candidate warehouses, and 5 customers. The data related to the costs of production, transportation, installation, and the amount of initial inventory are extracted from Tables 5 to 8 given in Petridis et al.^6^. The adoption parameter of blockchain technology is considered randomly in the interval^1–5^ based on the Likert scale. The probability of success of the attacker to manipulate the blocks is assumed to be 0.33.

**Figure 3:**
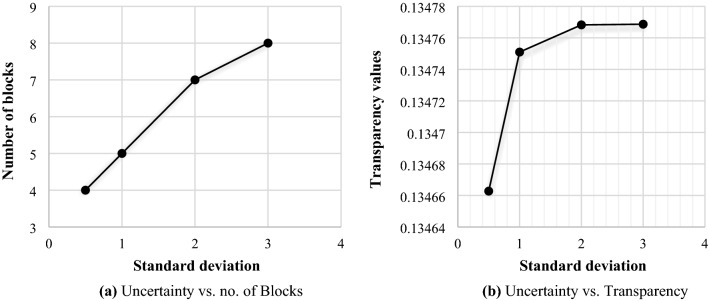
Relationship between uncertainty and blockchain technology.

Here, in addition to the number of blocks, the focus is on the amount of transparency obtained from the generation of blocks. It is noteworthy that increasing the number of blocks and level of transparency is not equivalent. By increasing the standard deviation of the random parameter, both the number of blocks and the amount of transparency increase. With the increase of blocks, the transparency also increases, so with the increase of each block, the amount of increase in transparency decreases. Figure 3a,b illustrate the increase in the number of blocks and transparency, respectively. It should be noted that these figures show that with the intensification of uncertainty, the number of blocks increases and more transparency is achieved for the supply chain. This result is remarkable for the supply chain manager in the sense that the supply chain aims to increase transparency to deal with increasing uncertainty.

**Figure 4:**
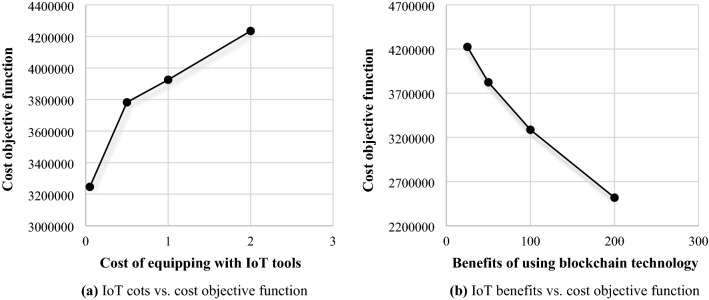
Relationship between blockchain adoption and cost objective function.

**Figure 5:**
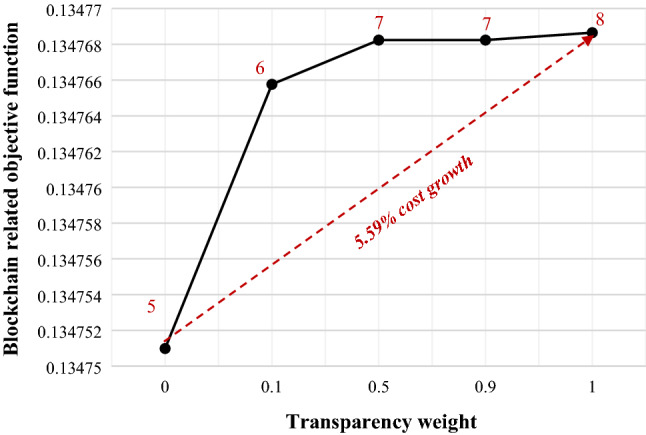
Relationship between cost, transparency and number of blocks.

In order to generate blocks, warehouses need to be equipped with IoT tools so that they can convert physical flow into blocks through digital flow. To equip with this tool, in our article, a fee is taken into consideration that is proportional to the installation cost. Other members who interact with the second layer (warehouses) benefit from being connected to the blockchain network. These benefits include tracking, transparency, security, better planning, and others. For this reason, these members are willing to provide a part of these benefits to equipped warehouses. Therefore, the benefits of using blockchain in the supply chain are assumed as a proportion of the fixed transportation cost between the warehouse and other members of the supply chain. Figure 4a represents that where the benefit factor is set equal to 100, with the increase in the cost factor of equipping warehouses with the IoT, the value of the cost objective function increases, while Fig. 4b shows that where the cost factor of the equipment is set equal to 0.1, as the benefit factor increases, the value of the cost objective function diminishes. Figure 5 displays the effects of the importance of objective functions against each other. By raising the weight of the objective function related to transparency, that is, the first objective function compared to the second objective function, the amount of transparency increases (the number of blocks increases from 5 to 8), while the supply chain costs may increase up to 5.59%. At the same time, it should be noted that the first-step increase in transparency (from 5 to 6 blocks) has caused the biggest change in the amount of transparency.

The important managerial conclusion extracted from these results is that supply chain managers can benefit from the adoption of blockchain technology when other co-members are willing to participate in the blockchain. In two cases, the suggested B&E algorithm is executed on the master problem based on the fuzzy programming presented in Eqs. (49)–(56). In Case 1, the costs and benefits of using blockchain in the supply chain are ignored, while in Case 2, these costs and benefits are considered. According to Petridis et al.^6^, the number of iterations of the algorithm is set to 2. The results are reported in Table 3. It must be noted that since FGP was executed to solve our proposed model, the values of the objective and membership functions corresponding to the iterations of B&E algorithm are reported in Table 3. As stated in the proposition, costs are reduced in the second iteration compared to the first iteration. On the other hand, due to the fact that the generation of blocks is dependent on the installation of supply chain members, the second iteration leads to a decrease in transparency. Compared to the first case, the second case cannot only reduce costs, but also create more transparency. Since the majority of research studies treat cost as an important criterion in the supply chain, thus, supply chain managers are recommended to use the developed B&E algorithm in the design of the supply chain because this algorithm is able to reduce costs by moving forward in iterations. In addition, supply chain managers using the first case can not only reduce costs but also ensure at least transparency in the supply chain. If supply chain managers can convince their partners of the benefits of using blockchain, by using the second case, they will both reduce costs and build more transparency for the supply chain compared to the first case. In this regard, it is worth mentioning that the amount of production in both the first and second cases is 25,000, but in the first case, the warehouses serve an average of 2.5 customers. However, the warehouses serve an average of 3.7 customers in the second case. It illustrates that in our investigation, the second case is more decentralized and customers receive service from more warehouses.

**Table 3:** Computational results of the improved B&E algorithm.

Costs and benefits of using blockchain technology	Iteration 1				Iteration 2			
	Transparency objective function (# of blocks)	Transparency membership function	Cost objective function	Cost membership function	Transparency objective function (# of blocks)	Transparency membership function	Cost objective function	Cost membership function
Case 1	0.13476873 (10)	0.909	3.97E+06	0.359	0.13476865 (8)	0.909	3.43E+06	0.636
Case 2	0.13476873 (10)	0.909	3.29E+06	0.538	0.13476872 (9)	0.909	2.98E+06	0.946

The efficiency values obtained from the SDEAT model are reported in Table 4. Accordingly, the efficiency values of warehouses in the first and second cases obtained by our SDEAT model are shown in Table 4. When the benefits and costs of using blockchain are not taken into account, 12 warehouses are efficient, while when such costs and benefits are considered, the efficiency of warehouses is improved and the number of efficient warehouses reaches 15. Therefore, supply chain managers are recommended to focus on the adoption of blockchain technology by supply chain members in order to enhance the efficiency of supply chain members.

**Table 4:** Efficiency of warehouses in the first and second cases.

DMUs	Case 1	Case 2	DMUs	Case 1	Case 2
1	1	0.999	11	1	1
2	0.938	0.938	12	1	1
3	0.944	1	13	0.952	0.983
4	0.97	1	14	1	1
5	0.98	0.98	15	1	1
6	1	1	16	1	1
7	0.976	1	17	1	1
8	1	1	18	1	1
9	0.928	0.96	19	1	1
10	1	1	20	0.957	1

**Figure 6:**
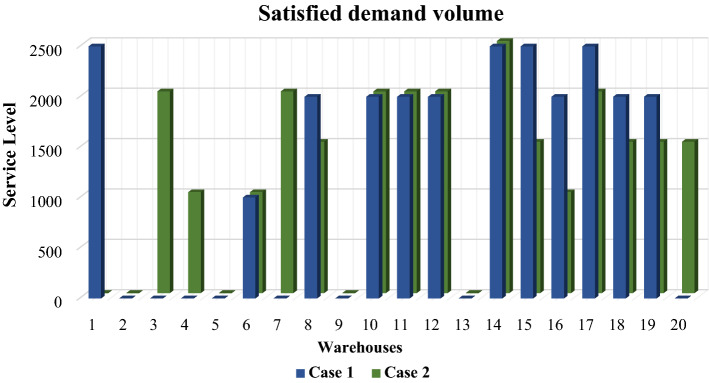
Satisfied demand volume by warehouses.

Figure 6 displays the volume of customer demand met by warehouses in the first and second cases. The dispersion of service between warehouses that are active in the first case is less than the dispersion of service between warehouses that are active in the second case. This shows that in the first case, the workload for the service is fairly distributed between warehouses. The second layer of the supply chain is connected to customers. Since the number of warehouses in the first case is small, the volume of demand satisfaction by each warehouse is high. In other words, this leads to traffic congestion of the goods and may delay the delivery time of the goods to the customer or disrupt the receipt of the goods by the customer. In this regard, supply chain managers are advised to benefit from the second case if decongestion is important to them, and from the first case if fairness among supply chain members is important to them.

**Figure 7:**
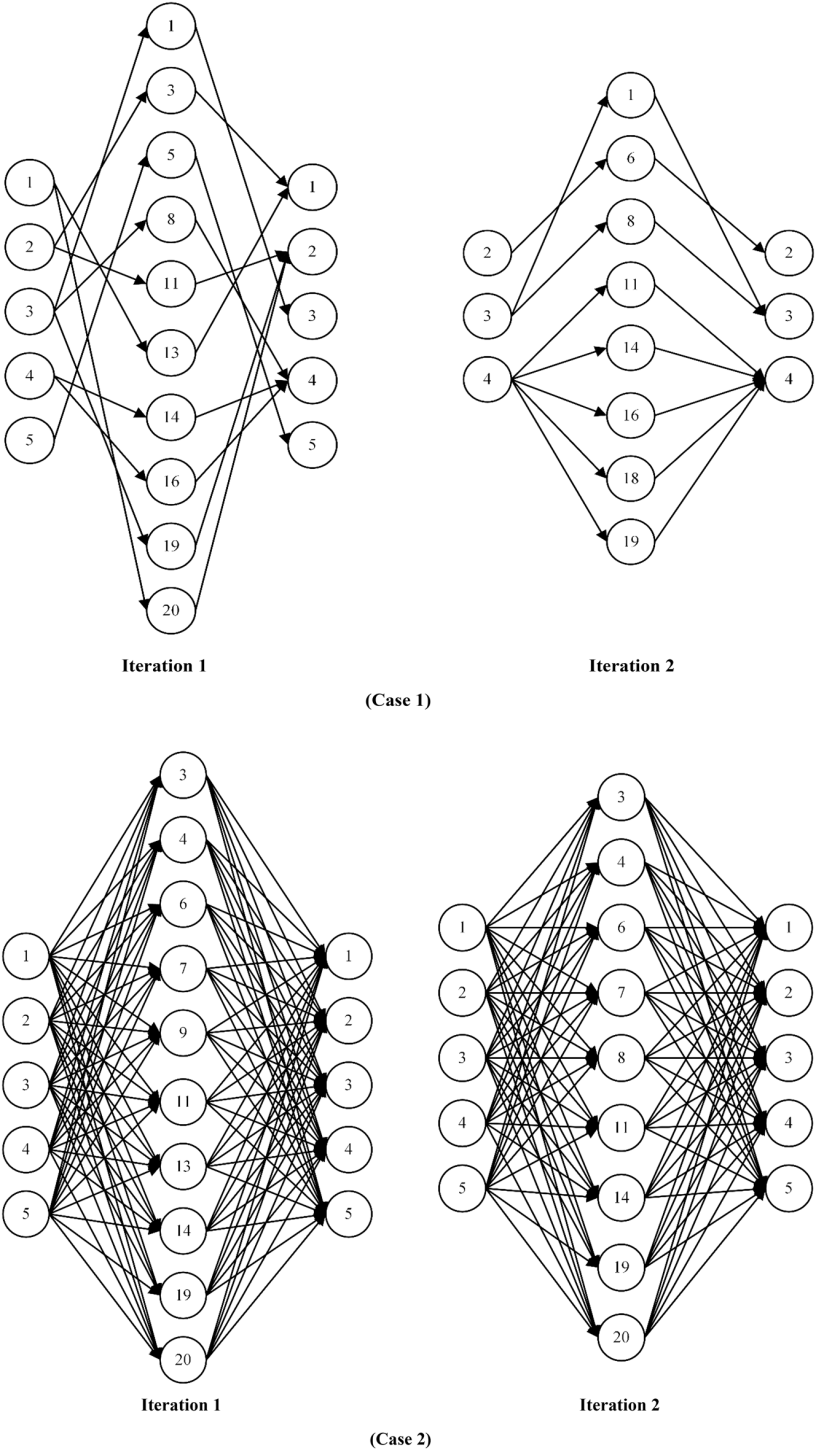
Members participating in the blockchain network.

Figure 7 outlines the members participating in the blockchain network in the first and second cases as well as the first and second iterations. Both for the first case and the second case, the participating members in the second iteration are fewer compared to the first iteration. The complexity, in terms of the number of participating members and the number of connections between them, is greater in the second case than in the first case. The first case provides the minimum necessary transparency while the second case aims to maximize the transparency. Therefore, it is recommended to supply chain managers; (a) If they work in an environment that is legislated for minimum transparency and they only want to follow the law, the first case is recommended, (b) if they are looking to maximize transparency, application the second case, (c) if they are looking for a higher level of security in the blockchain network, they need to use the second case due to its greater complexity, and (d) if they do not have advanced tools for blockchain-related computations, they should employ the first case.

In the second case, the negotiation power of supply chain managers is important to persuade members to join the blockchain. As mentioned in De Carvalho et al.^32^, blockchain is sometimes defined as the flow of information between levels of the supply chain. In this case, the establishment of the members of the supply chain requires a lower equipment cost because the activities of that member are not completely recorded in the blockchain. Accordingly, if the strategy of supply chain managers is cost minimization, the result of the second iteration of the second case can be improved. For this purpose, the term written in Eq. (57) is added to the cost Objective Function (2). In addition, the Constraints (16) and (17) are changed into Constraints (59) and (60), and Constraints (20) and (21) are changed into Constraints (61) and (62), where $\delta$ represents the cost factor of equipping the supply chain member to record the flow of information in the blockchain and ${\theta }_{j}^{DN}$ represents the member who records the flow of information in the blockchain. Constraint (58) shows the relationship between the blockchain members and supply chain. Constraint (63) guarantees that the member can only record information about flows or can only record a wide range of information about its internal activities in the blockchain.

57 $$\begin{array}{*{20}c} {{\text{minimize}}} & {\mathop \sum \limits_{j \in J} \delta f_{j}^{c} \theta_{j}^{DN} } \\ \end{array} ,$$

58 $$\begin{array}{*{20}c} {\theta_{j}^{DN} \le y_{j} } & \quad {\forall j \in J,} \\ \end{array}$$

59 $$\begin{array}{*{20}c} {B_{j}^{DN} + \theta_{j}^{DN} \le \mathop \sum \limits_{i \in I} B_{ij}^{1 \to 2} } & \quad {\forall j \in J} \\ \end{array} ,$$

60 $$\begin{array}{*{20}c} {B_{j}^{DN} + \theta_{j}^{DN} \le \mathop \sum \limits_{k \in K} B_{jk}^{2 \to 3} } & \quad {\forall j \in J,} \\ \end{array}$$

61 $$\begin{array}{*{20}c} {B_{ij}^{1 \to 2} \le B_{j}^{DN} + \theta_{j}^{DN} } & \quad {\forall i \in I;j \in J,} \\ \end{array}$$

62 $$\begin{array}{*{20}c} {B_{ij}^{1 \to 2} \le B_{j}^{DN} + \theta_{j}^{DN} } & \quad {\forall i \in I;j \in J,} \\ \end{array}$$

63 $$\theta_{j}^{DN} + B_{j}^{DN} = 1,$$

64 $$\begin{array}{*{20}c} \theta_{j}^{DN} \in \left\{ {0,1} \right\}& \quad {\forall j \in J,} \\ \end{array} .$$

**Figure 8:**
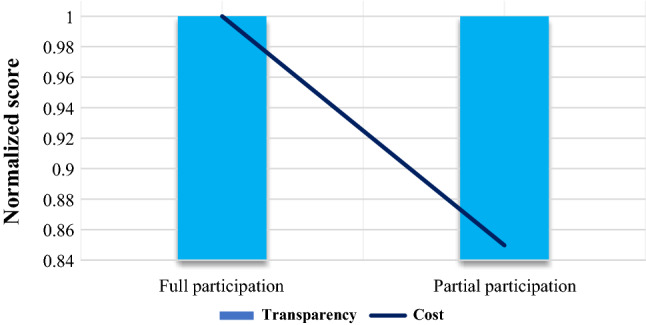
Partial vs. full participation of members in the blockchain.

Figure 8 compares the full participation of members and the partial participation of members (only information flow) in the blockchain with each other in terms of transparency and cost. Partial participation can significantly reduce costs while reducing transparency, although the reduction in transparency is much less than the cost reduction value. Therefore, considering partial participation can be suitable for managers who are very sensitive about the cost.

**Figure 9:**
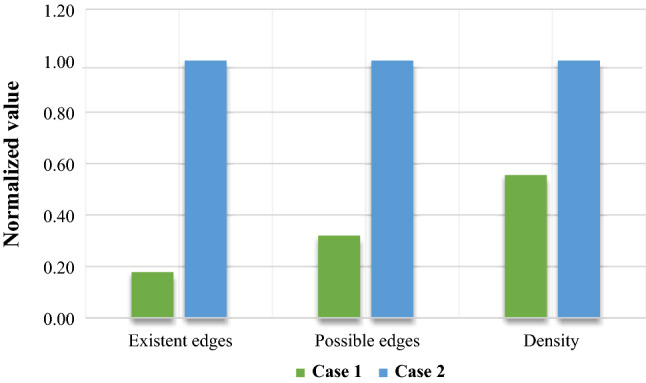
Case 1 vs. Case 2 based on density.

The participating members in the blockchain must verify the creation of the block through a consensus mechanism. Despite the fact that increasing the size of the network and the number of nodes can lead to transparency in this mechanism, the consumption of resources and time increases which can pose a challenge for blockchain under the name of scalability. Such an increase can lead to the density of operations related to blockchain technology and even damage the blockchain. To calculate the density of the blockchain network, the ratio of the number of edges of the blockchain network is utilized to the total possible edges of the blockchain network. Based on this, the blockchain network resulting from the first case and the blockchain network resulting from the second case are compared in Fig. 9. The numbers in this figure are reported as normalized numbers. Case two, compared to Case one, not only has a greater number of existing and possible edges, but also has a higher density.

A summary of managerial implications obtained from the results is outlined in Table 5.

**Table 5:** Goals of supply chain managers and recommended actions.

Goals and strategies of supply chain managers	Actions		
	Case 1	Case 2	Partial network
Dealing with uncertainty	✓	✓	
Reduced costs due to security, transparency and better planning		✓	
Following the law to ensure minimum transparency	✓		
Maximizing transparency		✓	
Minimizing SCD costs	✓	✓	
Improving SC member efficiency by negotiating with members to adopt blockchain		✓	
Decongestion in customer service		✓	
Observance of fairness between members for customer service	✓		
High level of security in blockchain (complexity)		✓	
Lack of access and provision of advanced tools for blockchain computations	✓		
Supply chain management focuses on supply chain cost and information flow			✓
Low level of density in blockchain (scalability)	✓		

It is worth noting that the current research differs substantially from the existing research on the blockchain adoption. Very important topics such as carbon trading between users in road transportation^48^, blockchain adoption in large-scale networks^49^, and the limitation of some blockchain characteristics (such as storage capacity)^50^, have been investigated in the field of blockchain technology in the research literature. Furthermore, blockchain members were addressed based on supply chain network, scalability, complexity and limitations of blockchain in this research. However, our method of investigation has differences from what have been done in the literature so far. The distinguishing point is that in the research literature, the blockchain adoption (e.g., in networks and supply chain) has been considered, while our study is thoroughly based on optimization models which are formulated according to operations research. Through these models, the supply chain is simultaneously designed and the blockchain is adopted on it. In this way, both the supply chain network affects the configuration of blockchain technology and the blockchain technology also affects the configuration of the supply chain network. Hence, decision-makers and supply chain managers may learn the application and advantages of our proposed models. In case of institutionalizing transparency in the supply chain at the same time as designing and planning the supply chain, the proposed models in this study are recommended.

Another difference between this work and the research literature is in decision-making levels. Our paper plans supply chain design and adoption of blockchain at the same time, while the research literature generally seeks to adopt and implement the blockchain technology in supply chain. Therefore, the supply chain managers in our view are involved in strategic and tactical decisions, while they are more involved in operational decisions according to the view of the research literature. All in all, according to the conditions of the supply chain, managers must pay attention to what level of decision-making they are amenable to adopt the blockchain technology in supply chain.

## Conclusion and outlook

Immutable sharing of information between supply chain members is an essential issue in the supply chain. For this purpose, the members of the supply chain should connect with each other in a transparent manner, and blockchain technology can bring such transparency to supply chains. This work aimed to integrate the three-level SCD with the adoption of blockchain technology. In this regard, for the first time in the literature, a bi-objective optimization model was proposed, which considers not only the costs of the supply chain (such as transportation, production, and installation) and IoT equipment related to blockchain, but also the transparency resulting from the adoption of blockchain technology in the supply chain. In addition, to the best of the authors’ knowledge, this study is the first attempt to address stochastic conditions for the minimum blockchain length. The proposed mathematical model is solved through the proposed B&E algorithm and FGP to generate both optimal and efficient solutions. In our article, the adoption of blockchain in SCD was investigated by comparing two cases. In the first case, only the transparency resulting from the use of blockchain was integrated into SCD, while in the second case, in addition to transparency, the cost and benefits resulting from the application of blockchain were addressed. The results revealed that the first case is superior in reducing computational complexity, creating fairness between service members to customers, and reducing scalability compared to the second case, while the second case is superior in creating more transparency, security, and efficiency of the supply chain members, and reducing congestion compared to the first case.

Consideration of blockchain in SCD through transparency has been neglected in research. In addition, there is not enough data on the implementation of blockchain in the supply chain and measuring the resulting transparency in the research literature. For this reason, the lack of access to similar articles and sufficient data is among the limitations of this study. Given that the examined subject is related to the use of cutting-edge technologies in SCD, this work can provide researchers with many research opportunities. Defining the application of blockchain through the combination of members and links simultaneously, considering the conditions of uncertainty in the measurement of transparency and other measurement related to blockchain technology, the adoption of blockchain in other supply chain structures are among the important innovations for future research.

## Data Availability

The datasets used and/or analyzed during the current study available from the corresponding author on reasonable request.
